# “I Believe That AI Will Recognize the Problem Before It Happens”: Qualitative Study Exploring Young Adults’ Perceptions of AI in Mental Health Care

**DOI:** 10.2196/76973

**Published:** 2025-08-25

**Authors:** Lena Petersson, Mikael G Ahlborg, Katrin Häggström Westberg

**Affiliations:** 1 School of Health and Welfare, Halmstad University, P.O.Box 823, Halmstad, 30118, Sweden, 46 7052055024

**Keywords:** anxiety, artificial intelligence, depression, primary care, mental health, young adults, qualitative, health care

## Abstract

**Background:**

Globally, young adults with mental health problems struggle to access appropriate and timely care, which may lead to a poorer future prognosis. Artificial intelligence (AI) is suggested to improve the quality of mental health care through increased capacities in diagnostics, monitoring, access, advanced decision-making, and digital consultations. Within mental health care, the design and application of AI solutions should elucidate the patient perspective on AI.

**Objective:**

The aim was to explore the perceptions of AI in mental health care from the viewpoint of young adults with experience of seeking help for common mental health problems.

**Methods:**

This was an interview study with 25 young adults aged between 18 and 30 years that applied a qualitative inductive design, with content analysis, to explore how AI-based technology can be used in mental health care.

**Results:**

Three categories were derived from the analysis, representing the participants’ perceptions of how AI-based technology can be used in care for mental health problems. The first category entailed perceptions of AI-based technology as a digital companion, supporting individuals at difficult times, reminding and suggesting self-care activities, suggesting sources of information, and generally being receptive to changes in behavior or mood. The second category revolved around AI enabling more effective care and functioning as a tool, both for the patient and health care professionals (HCPs). Young adults expressed confidence in AI to improve triage, screening, identification, and diagnosis. The third category concerned risks and skepticism toward AI as a product developed by humans with limitations. Young adults voiced concerns about security and integrity, and about AI being autonomous, incapable of human empathy but with strong predictive capabilities.

**Conclusions:**

Young adults recognize the potential of AI to serve as personalized support and its function as a digital guide and companion between mental health care consultations. It was believed that AI would function as a support in navigating the help-seeking process, ensuring that they avoid the “missing middle” service gap. They also voiced that AI will improve efficiency in health care, through monitoring, diagnostic accuracy, and reduction of the workload of HCPs, while simultaneously reducing the need for young adults to repeatedly tell their stories. Young adults express an ambivalence toward the use of AI in health care and voice risks of data integrity and bias. They consider AI to be more rational and objective than HCPs but do not want to forsake personal interaction with humans. Based on the results of this study and young adults’ perceptions of the monitoring capabilities of AI, future studies should define the boundaries regarding information collection responsibilities of the health care system versus the individuals’ responsibility for self-care.

## Introduction

### Background

Mental health problems such as depression and anxiety are common among the general population and often appear during adolescent years and early adulthood [1-3]. Recurring and extended episodes of mental health problems in adolescence lead to a poorer prognosis later in life, with increased risk of complex and persistent mental health problems [4]. Early interventions are advocated [5,6]; yet, globally, young adults with mental health problems struggle to access appropriate and timely care [7-10]. Artificial intelligence (AI) is suggested to be a potential part of the solution for mental health care.

The research argues that AI applications will improve the quality of health care through developments in diagnostics, monitoring, access, advanced decision-making, digital consultations, and AI-assisted interventions [11,12]. The anticipated potential is based on the ability of AI to process and learn from large volumes of data and to identify complex patterns. Combining data from multiple sources enriches AI analytic tasks, such as predictive modeling, by making them more data-driven and enabling an evidence-based practice 2.0 in health care [1,13] Thus, health care is one of AI’s most promising application areas [14]. Digitized data in various formats that can be analyzed are available in health care settings to create information-driven care [15]. Research has anticipated that AI and precision medicine have the potential to revolutionize health care by identifying patients with special care needs through insights and learning from complex data [16,17], supporting clinical work [18], increasing the efficiency and effectiveness of processes in health services [19], and supporting decision-making [20]. The digital transformation will give caregivers the opportunity to develop more accessible and personalized proactive care that is AI-powered.

However, the transformation of health care into information-driven care for mental health problems is complex and multifaceted and requires the feedback and involvement of both health care professionals (HCPs) and interest-holders [21], such as patients. AI research has primarily focused on the technical aspects that underlie the technology, whereas major challenges in applying AI to health care are embedded in its development, implementation, and use in routine care [22]. Empirical evidence of ways to succeed in implementing AI in health care is scarce and not in proportion to the current needs in practice [23]. In addition, care for mental health problems is a discipline that has been slow to adopt AI [24]. The discipline is unique in medicine, in that it relies on patients’ self-reports of their thoughts, feelings, symptoms, and social interactions. Clinicians must correctly interpret these reports to diagnose and treat, despite having varying levels of resources and skills. This implies that practices and decisions or recommendations may be highly subjective and vary considerably in appropriateness, quality, and efficiency [25].

New digital systems both enable and require collaboration between humans and technology. In this paper, AI is seen as an actor in a sociotechnical system [26]. The concept of an AI-powered health care is used to describe an information-driven infrastructure that supports the shift from decision-making based primarily on human knowledge to information-driven decision-making supported by various forms of AI. Clinicians working in mental health care will likely benefit greatly from AI, given that it seems to possess the capacity to accurately detect and predict mental health conditions and can thus guide diagnostic work and provide information on alternative treatment methods [11]. AI is also thought to enhance person-centered care [27].

Due to their familiarity with using digital technology in various aspects of daily life, young adults are frequently identified as a patient group for whom technological interventions may be most readily implemented [28]. It is important to incorporate the views of the interest holders because help-seeking for mental health problems and navigation of the health care system are dynamic and challenging processes, encompassing both individual problems and aspects of the health care organization within societal structures [29]. Elucidating the perceptions of young adults will help ensure that implementation aligns with the target group’s values, needs, and concerns. Furthermore, acknowledging barriers to acceptance may facilitate sustained engagement and improved treatment compliance, which is critical in an otherwise strained health care context. While there is some research elucidating the patient perspective, the perceptions of young adults of AI in health care remain relatively underexplored [30], particularly within the context of mental health services. Except for limited participatory studies focused on chatbot development [31-33], this area remains largely underexamined. Such research is essential to allow for insights into how perceived organizational factors within the health care system may influence help-seeking behaviors and the delivery of timely and accurate care. Given this background, this study seeks to explore the perceptions of young adults on AI-based technologies within mental health care. This understanding is essential for developing strategies to address potential challenges in AI development and implementation and to optimize the use of AI applications in mental health care.

### Aim

The aim was to explore perceptions of AI in mental health care from the view of young adults with experience of seeking help for common mental health problems.

## Methods

### Study Design

This study used a qualitative inductive design with conventional content analysis [34] of individual interviews. The interviews explored how AI-based technology can be used in mental health care from the viewpoint of young adults with experience of seeking help for common mental health problems. This approach allowed for a systematic and inductive identification of categories that emerged from the data. To ensure trustworthiness, the study is in accordance with the COREQ (Consolidated Criteria for Reporting Qualitative Research) 32‐item checklist [35] (Checklist 1).

### Settings

The Swedish health care system is publicly financed. Health care responsibility is decentralized to 21 county councils, whose responsibilities include health care provision and the promotion of good health for citizens. Young adults with mental health problems may turn to a variety of health care settings, of which primary care is the “first line care,” while specialist psychiatric care is reserved for more severe cases of psychiatric illness [36]. Students at Swedish universities may also turn to the student health care system. These centers offer counseling sessions at no cost to students regarding their health and well-being. This interview study was conducted at a university in the south of Sweden and, although recruitment was done through the student health care center, many of the participants also had previous care experiences from other health care settings.

### Participants

Young adults between the ages of 18 and 30 years seeking help for common mental health problems from a student health care center at a university in southern Sweden were invited to participate in the study. Health care staff at the center aided in the recruitment phase. They informed the young adults about the study and the opportunity to participate and handed out brief written information about the study. All young adults indicated whether they were interested or not in participating by placing a signed written form in an envelope, sealing it, and handing it to the center staff. The staff then sent the sealed envelopes to 1 author (LP). Thus, the staff were unaware whether the students had accepted or declined participation in the study. In total, 25 young adults were interviewed: 16 women and 9 men, aged 20 to 30 years. All participants had previous experience of seeking help for mental health problems; however, both the extent of their previous experience with AI tools in mental health care as well as their general knowledge on AI were unknown, and the researchers did not specifically ask for this information.

### Data Collection

The individual, semistructured interviews were performed at the university campus or by video call (using either the Microsoft Teams or Zoom [Zoom Video Communications] platforms) between December 2021 and January 2023 either by researcher LP or Susanne Lindberg. In total, 17 interviews were conducted at the university campus, and 8 were conducted on the web. The researchers had not met the students before the interviews in any professional or student-teacher settings. Every interview started with the introductory question, “Can you tell me about your experiences of seeking care for mental health problems?” They were then asked to describe their experience of digital technology during help-seeking, care, and treatment for mental health problems. They were also asked to elaborate on the capability of AI to predict, treat, and support them with mental health problems and any changes that could be foreseen due to the implementation of, and use of, AI in mental health care. Each interview ranged between 19 and 67 minutes, and the total time was 17 hours and 2 minutes, with a mean of 41 (SD 11.55) minutes per interview. The interviews were audio-recorded and transcribed verbatim. The recruitment of participants ended when saturation was reached.

### Data Analysis

A conventional content analysis was used, in accordance with Hsieh and Shannon [34], to suit the data and the aim of the study. The method was chosen due to the relative novelty of the subject, with limited previous research, a situation that called for an inductive and open approach to data. As knowledge from content analysis is grounded in data, this aids in gaining and developing information from study participants without imposing a preconceived theoretical understanding [34]. To enhance the validity of the findings, investigator triangulation was used. The 25 transcriptions were read multiple times by the 3 authors, LP, MA, and KHW, to achieve immersion in the material and gain an overview of the full dataset. The authors have extensive experience in research about mental health and qualitative methodology. Subsequently, the interview data were divided among the 3 authors who coded the allocated interviews separately. The interviews were distributed randomly among the 3 authors. No pre-established frameworks were applied, as codes were generated directly from the data. The authors had research meetings after the first inductive coding to discuss thoughts and an initial analysis. Further coding was then done by each author individually, after which, meetings took place to discuss and formulate emerging and tentative codes. The authors, in conjunction, grouped and organized the data into subcategories and categories based on how the various codes related and linked. The subcategories and categories were extensively discussed among the research team, with adjustments made until consensus was reached. Categories were chosen as the highest level of abstraction. To illustrate the findings, appropriate quotations from the data were chosen by the research team, translated into English, and in some cases slightly amended to enhance comprehensibility, but the essence and meaning of the participants’ responses were preserved in detail. The translations were further checked by a professional translator for accuracy and a true representation of the original statements.

### Ethical Considerations

The Swedish Ethical Review Authority (2020‐06246) approved the project. Participants signed an informed consent before entering the study. Participation was voluntary, and participants could withdraw at any time without stating a reason. Participation in the study did not in any way affect their treatment, and there was no compensation for participation. The study adhered to the guidelines in the Declaration of Helsinki [37].

## Results

### Overview

**Figure 1.**
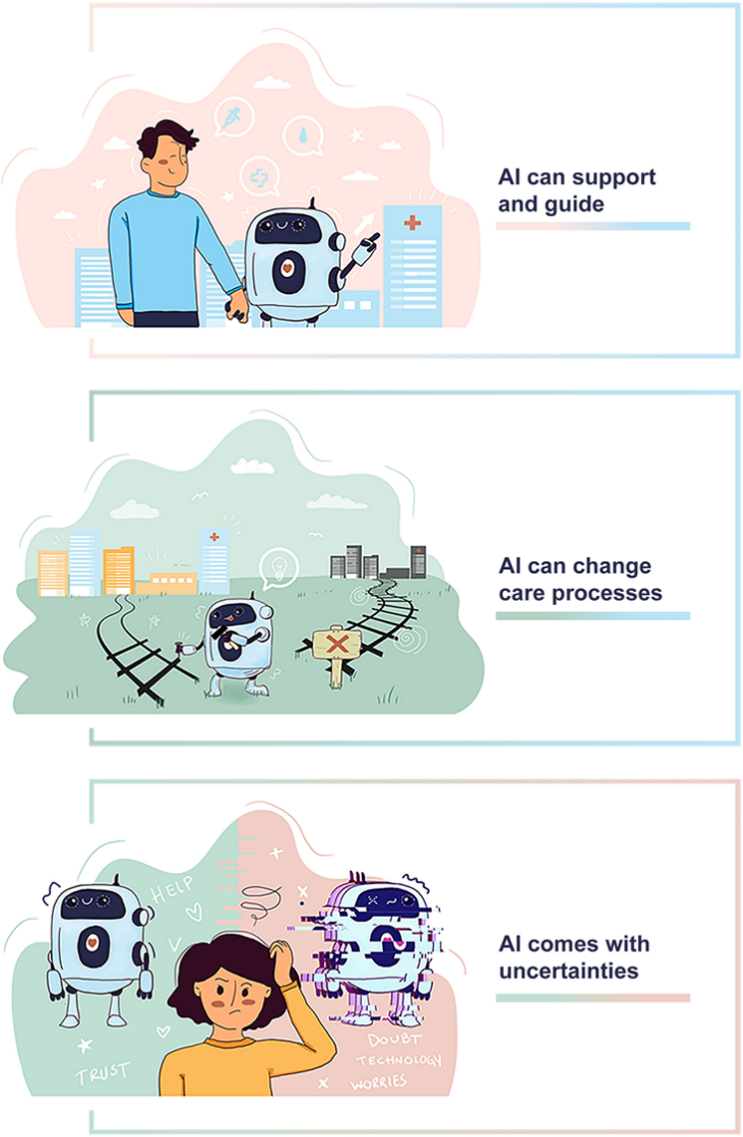
Categories. AI: artificial intelligence.

Three main categories were derived from the analysis of the data (Figure 1). These categories represent the participants’ perceptions of how AI-based technology can be used in mental health care.

### AI Can Support and Guide

Young adults perceived that AI-based technology could offer navigation support in the health care system. They expressed uncertainty about places to turn for common mental health problems and difficulties in assessing one’s own problems. They described that AI would play a role in personal decision-making and give guidance on when, how, and where to contact HCPs. AI was also described as potentially becoming a digital companion that could look out for you in various ways. A digital companion would, for example, be there for you if you wanted to chat with someone, remind you about self-care activities, suggest sources of information, and be receptive to changes in behavior or mood.

*Or perhaps it might recommend things, or, yeah for example that you can turn to different ... places for example to seek care if you needed to. Or that it can ask you if you would like to chat with a real person, or just ... or if you just need to talk someone really ...*

[Interview 21]

Among young adults, there was a perception of AI as a potential recipient of self-reported data. They believed that, by using this data, AI would be able to outline, follow, and present trends, ask follow-up questions, and “nudge” patients in various directions. For some, this meant that AI would be able to empower patients by offering visual summaries or insights into their recent mental health state. Based on the AI-generated feedback and summaries, AI could pose follow-up questions tailored to individual experiences, suggest relevant sources of information, or recommend contacting an HCP when necessary. Notably, young adults perceived that they would be susceptible to this form of “nudging,” irrespective of the specific suggestions made by the AI.

*Instead of going on Google and playing hypochondriac, you could have an AI developed by healthcare personnel that could ... well, sort of like “it could be this and this” and then you can ask some questions, and then you would get a “yes, but there is nothing to worry about, or you should visit your healthcare centre.”*

[Interview 9]

Based on self-reported data, young adults recognized the potential of AI to recommend personalized self-care activities. In this scenario, HCP could prescribe an AI chatbot to coach patients for short time periods while assessing the need for additional care. It was suggested that such a tool could enhance patients’ sense of validation and reduce the necessity for early pharmaceutical prescriptions.

Young adults believed that AI could play a valuable role in supporting patients through difficult episodes. With AI being constantly available, patients would have immediate access to conversational support. This was described as particularly beneficial in times of distress, where normally there would be waiting times for human support.

*They are not physically there, but it can assist until help arrives, and if you have a panic attack and you may not know what it is, for example, then you will be very scared. If you have one then ... Call 1177 [Information line on illnesses, care and health], there is a waiting time of, say, 20 minutes. But do these steps. Then AI will be there, almost holding your hand, helping you along the way. It does nothing in itself, but it still helps to hear “do this” or things like that. Just to calm the situation down. Because I think that’s a lot of what it’s about, that you stress yourself out.*

[Interview 3]

Young adults also described great potential in AI to provide companionship during moments of loneliness or when seeking advice. This could be especially valuable between therapy sessions or when a treatment period has ended, and the young adult just wants to “check in” with someone. It was expressed that reaching out to a chatbot presented a lower barrier compared to scheduling a regular therapy session and resulted in less burden on the health care system. Additionally, they expressed the idea of an AI chatbot proactively checking in on them, similar to how a close friend might reach out to ensure that they were doing okay.

*Yes, but I know, at least from my own experience, that it’s been quite a long time since I had any contact with healthcare. And, perhaps, I have been starting to feel worse again or what you call it ... on the one hand perhaps it [AI] checks in, in some way, on how you are. Or that you get some sort of ... yeah, “how is it going now?”*

[Interview 21]

In reference to in-person therapy sessions, some of the young adults described negative thoughts arising only a couple of hours after sessions. In these cases, it was believed that an AI chatbot could “be there” for them in more acute situations as well and that AI would be sensitive and adapt to individual preferences and needs based on previous interactions.

### AI Can Change Care Processes

This category revolved around how AI could enable more effective care processes, functioning as a tool for both patients and HCPs. Young adults expressed confidence in AI to improve triage, screening, identification, and diagnosis. AI was seen as having the potential to discern patterns and symptoms of mental health problems and communicate them back to both patients and HCPs.

*And then there is someone who can read that “yes, but these three things ...” or “this is now in place. Now we need to somehow get in touch with this person.”*

[Interview 10]

By using AI as a screening and identification device, it was assumed that this would shorten health care trajectories and the help-seeking process. AI could “sound the alarm” and identify those who were progressively developing more severe mental health problems. There was great optimism about the potential of AI to help initiate health care consultations before people got “really sick.” In some cases, young adults described how the information on an individual’s mental health state would be passed on to the HCP for them to act upon. It was rarely expressed how this would happen and by what means AI would be able to monitor and collect information on a person’s mental health status. However, belief in AI’s potential to discern patterns and symptoms was high.

*I believe that AI will recognize the problem before it happens.*

[Interview 12]

AI was also seen as being able to communicate a person’s mental health problems and symptoms to the HCP. Young adults pictured AI as something that could gather and summarize information and provide the relevant information back to the HCP during and in between visits. AI was also believed to be able to function as a diagnostic tool, as it has the ability to process more information. Thus, AI was thought to assist HCPs and present treatment suggestions. Trust in AI was high, as it was thought that AI could process more information than humans, provide an accurate diagnostic picture, and help HCPs become aware earlier of an underlying problem by discovering patterns and symptoms.

*Not everybody believes in this, and a lot of people doubt the technology and are against it, but I believe it will help and take some weight off the shoulders of a doctor or a counsellor. It will help them get to a diagnosis or solution faster and help those who really need it.*

[Interview 8]

It was also believed that AI had the potential to reduce waiting times through a more accurate screening and prioritizing process, ultimately resulting in safer care. Young adults described how AI would be used to summarize and provide information to HCPs, so that they did not have to reiterate the same information to various HCPs.

This swift information transfer also meant that treatments would be initiated closer to the first visit. Between visits, AI was seen as potentially helpful in gathering relevant information from the patient and sending it to the HCPs. Pertaining to this was the impression that, because AI was both smarter and faster than humans, the need for HCPs, primarily physicians, would decrease. Young adults described that AI would process more information than humans, and in a rational and objective manner. This was thought to be a good thing, as AI was portrayed as rational and nonjudgmental on delicate matters, such as mental health problems and help-seeking.

*For me, at least, often it was like, “Okay, I’m not feeling so great mentally,” and it took a while before I dared to seek help. So, it would have been really nice, if ... Because you know that it’s not a human, when it’s an AI, so you can ... as soon as you talk to a human, you’re a little afraid of being judged or something. So, an AI is good in that way, that it’s completely impartial, or non-human.*

[Interview 9]

### AI Comes With Uncertainties

This category concerned the uncertainties and risks that young adults perceived to be associated with AI technology in the health care setting, based on their experiences of mental health problems. Young adults expressed uncertainties related to AI both as a “product” developed by humans, entailing limitations and security and integrity concerns, and as an autonomous entity, incapable of human empathy but with strong predictive capabilities.

As a human product, AI was believed to constitute a risk to personal integrity. Young adults voiced concerns about the amount of data that could be collected and the extent to which personal information should be investigated, assessed, and interpreted. The issue of safeguarding anonymity and current data protection legislation was raised. Given that it is connected to the internet, AI was deemed to be an unsafe milieu, and data security unattainable.

*I believe that everything that is connected to the internet can be hacked, nothing can be a 100% safe.*

[Interview 12]

This was contrasted to the safety of personal, one-to-one meetings that were depicted as safer. Going to a personal meeting with an HCP was expressed as installing a sense of security in contrast to consulting AI, which could not be trusted with information.

*It’s more that you don’t trust what the internet does with one’s information and therefore, going to the youth clinic and meeting with someone for a talk, that feels safer.*

[Interview 13]

Young adults also saw risks concerning the information sharing aspect and named issues that information can be misused and that it was uncomfortable and daunting to share personal information about one’s mental health. They also voiced concerns about the role of AI in society and the sheer amount of information that AI collects in various settings and has access to. This was seen as ethically problematic, as the control of information was uncertain, and there was a risk of misuse.

*Even if we are fairly unaware of it, for example that Instagram and TikTok retain all our information, I think that ... well, mapping information is difficult. Who is going to have that information ... I’m not sure.*

[Interview 24]

Another concern was that AI was ultimately designed and influenced by humans, and the possibility of flawed algorithms was apparent. This was described as a risk of human biases tainting the process.

*It’s still people that programme AI and, somehow, we transfer our prejudice and our ways of thinking to AI ... So, I think this is something one needs to consider, how we as people influence how AI thinks.*

[Interview 24]

The outcome of AI as a human product was also described as limited due to the amount and nature of the information the AI had access to. Simultaneously, AI was described as more adaptive and impersonal toward patients. Young adults held the view that AI could only explore and interview a person in a standardized manner and that a physical person had a greater ability to adapt to the person in need. Thus, some young adults considered the use of AI to be limited, as AI would not be able to interact empathetically, nuanced, and intuitively as a physical “helper.” Young adults described that the “best help” they had received was because they “clicked” with someone. Efficient help was thus seen as having a personal component and being situated at a relational level—and you cannot have a relationship with AI. It was voiced that AI would become too objective and too rational and therefore would miss the note of humanness. As AI was seen as a very technical entity, it was not able to replace human compassion. Human contact was emphasized as being a vital component in recovery and treatment for mental health problems; thus, the trust in AI replacing human relationships was very limited.

*If you feel bad mentally, you don’t want to talk to a robot. You won’t see a robot and talk about your mental health because it doesn’t care ... So, I would say that it’s darn personal, and between two people when it comes to these things.*

[Interview 14]

From another perspective, young adults expressed skepticism about AI’s capacity to act as an autonomous being without the influence of humans. Given the inherent nature of mental health, AI would not be sufficient to disentangle the complexity of mental health problems on its own. Young adults voiced that AI could only act based on existing information and that AI would not be able to select the most relevant information. Impressions were that AI would not make well-balanced decisions, as its algorithms are incomplete. AI would not have access to nor would it be able to process all information, particularly since mental health problems are complex and vary in their nature. Young adults thought that AI would not see nuances in their mental health, nor would it have the capability to read between the lines.

*But when it comes to mental illness, I think it’s very difficult to determine what is what. And I don’t really think that the ... an AI robot can fee- ... get into the human psyche in the same way. Because it’s still about the concrete feeling. A human can’t understand their own feelings. So, how can a robot do that?*

[Interview 23]

AI was also portrayed as something that posed a risk of medicalizing common mental health problems. Young adults mentioned that AI will potentially trigger thoughts of more severe mental health problems and disorders, thus increasing anxiety instead of having a supportive function. Some expressed that the capability of AI to predict and foresee mental health problems would have a self-fulfilling effect.

*It might have made things worse too, because you would think, "Well, it doesn’t matter what I do today, because in five months it said I would have anxiety and then it becomes a bit like, “It said it and then it will happen”, no matter what I do, it will happen.*

[Interview 3]

Besides the belief that AI would enhance diagnostic procedures and treatments, there was also a concern that AI would diagnose incorrectly. Some young adults expressed a concern about the margin of error in AI and the ethical considerations in helping many but still harming some when AI could not diagnose correctly. Thus, it was posed that a diagnostic procedure with the aid of AI had to be extremely thorough to guarantee that no mistakes were made. Generally, young adults thought that the benefits of using AI for diagnostic procedures outweighed the risks. They often concluded that, by paying respect to details in the diagnostic procedure, AI could limit the errors, but there was a risk of relying too much on it. Young adults also voiced that the ultimate responsibility for health care decisions still rested with the professionals.

Although the use of AI in many instances was regarded as generally positive, with some skepticism, it was not foreseen as remedying the lack of resources in mental health care and staff shortages. Technical illiteracy and language barriers were mentioned as affecting the possibility of accessing care. In summary, young adults harbored an ambivalence toward AI. Both risks and benefits were described in relation to the technical apparatus, the adaptation to human needs, and in terms of prediction and decision-making capabilities.

## Discussion

### Principal Findings

This study sought to address a gap in the literature by exploring the broad perspectives of young adults with lived experience of mental health problems regarding the use of AI in mental health care. The principal findings showed that young adults perceived that AI had the potential to support and guide before the first contact with health care and between visits. AI was also thought to be able to change and enhance care processes. Alongside these positive expectations, young adults voiced an ambivalence, expressing uncertainties about the technology, but also acknowledging AI’s potential to ameliorate care for common mental health problems.

The results showed that young adults expected that AI would support them in navigating the health care system. AI was thought of as a possible contributor toward personalized help-seeking in the health care system and personalized support and treatment from HCPs. It has consistently been reported that seeking help for common mental health problems is a challenge for young adults [7-10]. The health care system is fragmented and, despite continuous efforts to enhance mental health care, the challenges seem to persist. This has been referred to as “The missing middle service gaps,” which describes a gap in mental health care, where the existing needs of individuals are not met by the health care system [38]. Interestingly, the structural issue of the health care system was not addressed as such; instead, AI was suggested to be able to aid and support young adults in navigating the fragmented health care system.

Another area where young adults saw the potential of AI was described as AI functioning as a digital companion in the time between consultations with health care. Young adults perceived this time “between mental health care visits” as a challenge, as they had limited knowledge of mental health issues and self-supporting activities. Recent research has shown both the usefulness of generative AI chatbots on matters associated with mental health [39] and how issues pertaining to trust are raised [40]. For AI to function as a tool and a digital companion, AI systems would have to adequately capture what is desired, that is, both emotional and sensitive matters and practical guidance; this requires good tonality of AI [41].

In this study, young adults were convinced that AI would enhance the efficiency and accuracy of mental health care. AI was thought to be able to summarize information during and between care visits and increase diagnostic accuracy. AI was also described as being able to shorten health care journeys, detect deterioration, and alert HCPs. Previous reviews highlight that AI has the potential to support clinical work [18,42], increase the efficiency and effectiveness of processes in health services [43], and enhance decision-making [20]. Thus, young adults correctly identified the need for AI-informed mental health care with improved diagnostic accuracy and treatments. The capability of AI to process larger amounts of information suggests that the time between initial symptoms, diagnosis, and treatment could be reduced [42] and holds promise as a tool for democratization of mental health care, with quicker and more efficient care [44]. Considering the challenges that many young adults with mental health problems face in accessing care [10], AI might play an important role, contributing to improving their experiences. Young adults did not express many thoughts on issues pertaining to personalized medicine nor tailoring treatments. Rather, they consistently stressed the organizational aspects, efficiency, and availability. This puts focus on how AI within the health care system can encompass both young adults and HCPs and not only the organizational but also the technical aspects of the system.

Thoughts on AI continuously monitoring well-being were brought up by young adults, who described AI as proactively checking in on them. Such an AI chatbot was envisioned as prescribed by HCPs and that the AI would enable both young adults and HCPs to act upon the received information. Recent research shows promising results that align with the views of young adults in this study on the potential of AI in preventive and personalized care [42,43]. AI can be used, and is used, as a method for monitoring the mental health status of patients through various systems, such as wearables and applications on smartphones [44]. For example, passively collected data may detect mood episodes in individuals with bipolar disorder with a high level of accuracy [45]. However, screening and monitoring in a general population present challenges, not least ethical, regarding trust and responsibility. Specific thoughts and issues surrounding ways in which monitoring should be carried out were not brought up by young adults in this study. However, they raised issues relating to the capacity of the health care system and how an increased demand for health care would be met.

Regarding uncertainties, young adults expressed an ambivalence toward AI. They voiced a concern for losing the “human touch” with the implementation of AI but simultaneously acknowledged that there might be benefits in several mental health care areas. This aligns well with the findings of Fazakerley et al [30], where concerns were raised regarding the lack of empathy in interactions with AI. Considering the need of young adults for trusting relationships when accessing support for mental health problems in primary care [46], the concern about AI’s empathic qualities is valid. Additional apprehension voiced by young adults in this study surrounded AI’s potential to perpetuate bias. AI as a tool in mental health care is trained on inherently biased datasets, and thus runs the risk of preserving, and possibly even aggravating bias, ultimately contributing to inequality in mental health care [44].

Potential risks that could arise from data collection in conjunction with AI use and privacy aspects have been acknowledged both by studies of HCPs [30] and in systematic reviews [42,44]. In this study, young adults brought up privacy issues relating to data containment and dissemination risks in conjunction with the use of AI. Issues of unauthorized access and data leakage are considered to be of utmost concern in the future use of AI [44]. They described feeling safer meeting professionals on site rather than using AI for support with mental health problems. This highlights an interesting detail, given the recent developments of AI-powered advanced speech-to-text technology, where the conversations during visits are recorded and summarized by AI. Such technology is becoming increasingly used in an effort to streamline clinical workflow, reduce dictation hours, and improve document legibility [47]. Thus, even at physical appointments on-site, AI will be present, collecting information. Ensuring transparency and data security is crucial in the future implementation of AI in the health care system [42]. Although young adults in this study voiced concerns regarding the safety of information-sharing with respect to AI, the notion of responsibility did not come up, that is, who and where in the health care organization the responsibility will reside for accessing, surveying, and acting on the information gathered by AI.

Future research on the responsibility and boundaries of mental health care is needed. Based on the perceptions of young adults of continuous monitoring, it is important to define and distinguish between the responsibilities of the health care system and the individual. What are the differences between AI-prescribed support and AI tools that young adults initiate themselves? Will the development of AI change the views on when, where, and how individuals turn from young adults into patients? Another relevant issue is related to screening capabilities. If AI-assisted prediction can increase the number of identified individuals with mental health problems, what will this entail from the resource-based and ethical perspectives?

### Strengths and Limitations

Within qualitative research, factors such as credibility, dependability, confirmability, and transferability are used to define trustworthiness [48]. The sample consisted of young adults who had previous contact with student health care at a university in southern Sweden. They were diverse in terms of gender and experiences in seeking mental health support, which enhances the credibility of the study. Transferability may be limited, given how participants were recruited from one single university. However, students at the university come from a diverse background, which implies a broad representation of the study sample. Moreover, the researchers’ expertise in the methodology facilitated thorough interviews, which stands as an additional strength of the research. To strengthen dependability, a semistructured interview guide was used, ensuring consistency in the questions asked (Multimedia Appendix 1). Once voluntariness was ensured, young adults had the option to conduct their interviews either in person or remotely via Microsoft Teams or Zoom. This variation in interview settings introduces potential methodological limitations, as differences in format may influence data consistency and, consequently, the study’s credibility. However, remote interviews using videoconferencing platforms like Zoom or Microsoft Teams can also be advantageous and are sometimes preferred by research participants [49].

Throughout the study, researchers maintained a nondirective approach, refraining from questioning or challenging the perspectives of young adults during interviews or subsequent analysis. This methodological choice was deliberate, as the primary objective was to accurately capture and convey the participants’ viewpoints on AI within mental health care in general. The young adults had varying levels of AI knowledge and experience, which required a cautious approach to interpretation and abstraction during the analysis. Participants may have had contact through AI tools in health care knowingly but also unknowingly, as AI tools are sometimes used in health care settings without patients being specifically informed about them. The span of knowledge on digital technologies and AI among participants was wide. Some expressed good knowledge mainly referring to their field of university studies, whereas others claimed to be fairly ignorant on these matters. This means that participants’ expressed views on AI in health care for mental health may be based on direct experience, informed knowledge, or in some cases, speculative imagination. By keeping the analysis on a more descriptive level, the study aimed to provide an authentic and transparent representation of their perceptions. To enhance confirmability, selected quotations from the data were incorporated to illustrate and substantiate the perceptions of young adults.

### Conclusions

In this study, young adults recognized the potential of AI to serve as personalized support, functioning as a digital guide and companion between mental health care consultations. AI was suggested to be able to aid and support young adults to navigate the help-seeking process, ensuring that they avoided the “missing middle” service gap. AI was thought to improve efficiency in the health care organization through pattern detection and monitoring, shortening wait times, improving diagnostic accuracy, and reducing the workload on HCPs while simultaneously reducing the need for young adults to repeatedly tell their mental health stories at every health care encounter. Young adults expressed an ambivalence toward the use of AI in care for common mental health problems. Even though AI was thought to be more rational and objective than humans, young adults did not want to forsake personal interaction with HCPs. Based on the participants’ perceptions of AI’s monitoring capabilities, future studies need to define the boundaries surrounding the information collection responsibilities of the health care system. A clarification on when mental health problems transform from being the individual’s responsibility for self-care to becoming a responsibility for the health care system would be beneficial for both the system and the individual.

## Supplementary material

10.2196/76973 Multimedia Appendix 1 Interview guide.

10.2196/76973 Checklist 1 COREQ checklist.

## Abbreviations

AI: artificial intelligence
COREQ: Consolidated Criteria for Reporting Qualitative Research
HCP: health care professional
