# Supplementary material for: “I Believe That AI Will Recognize the Problem Before It Happens”: Qualitative Study Exploring Young Adults’ Perceptions of AI in Mental Health Care
Source: JMIR Ment Health. 2025 Aug 25;12:e76973. doi: 10.2196/76973 (PMC12377516; doi:10.2196/76973)
Supplement: Multimedia Appendix 1 [file mental-v12-e76973-s001.docx]

Are you familiar with AI?

How do you see healthcare being able to identify (through AI analyses) and support the young adults with mental illness who are most in need of help and who are at risk of developing a psychiatric diagnosis? What would need to be different compared to how it is today?

How do you think that AI technology as a decision support to predict mental illness could be used in relation to this care process?

Do you see any unexpected and/or unavoidable consequences that may arise from using AI technology as decision support to predict mental illness?

How would you define success in relation to how AI technology as decision support can predict mental illness and provide direction in developing preventive interventions?
